# Geographic Mobility and HIV Care Engagement among People Living with HIV in Rural Kenya and Uganda

**DOI:** 10.3390/tropicalmed8110496

**Published:** 2023-11-10

**Authors:** James Ayieko, Marguerite Thorp, Monica Getahun, Monica Gandhi, Irene Maeri, Sarah A. Gutin, Jaffer Okiring, Moses R. Kamya, Elizabeth A. Bukusi, Edwin D. Charlebois, Maya Petersen, Diane V. Havlir, Carol S. Camlin, Pamela M. Murnane

**Affiliations:** 1 Kenya Medical Research Institute, Center for Microbiology Research, Nairobi 00200, Kenya; 2 Division of Infectious Diseases, David Geffen School of Medicine, University of California Los Angeles, Los Angeles, CA 90024, USA; 3 Department of Obstetrics, Gynecology & Reproductive Sciences, University of California, San Francisco, CA 94143, USA; 4 Division of HIV, Infectious Diseases and Global Medicine, Department of Medicine, University of California, San Francisco, CA 94143, USA; 5 Department of Community Health Systems, School of Nursing, University of California, San Francisco, CA 94143, USA; 6 Clinical Epidemiology Unit, School of Medicine, College of Health Sciences, Makerere University, Kampala P.O. Box 7072, Uganda; 7 School of Medicine, Makerere University, Kampala P.O. Box 7072, Uganda; 8 Center for AIDS Prevention Studies, Department of Medicine, University of California, San Francisco, CA 94143, USA; 9 Division of Biostatistics, University of California, Berkeley, CA 94720, USA; 10 Department of Epidemiology and Biostatistics, University of California, San Francisco, CA 94143, USA; 11 Institute for Global Health Sciences, University of California, San Francisco, CA 94143, USA

**Keywords:** HIV, human mobility, retention in care, treatment adherence, sex differences

## Abstract

Introduction: Human mobility is a critical aspect of existence and survival, but may compromise care engagement among people living with HIV (PLHIV). We examined the association between various forms of human mobility with retention in HIV care and antiretroviral treatment (ART) interruptions. Methods: In a cohort of adult PLHIV in Kenya and Uganda, we collected surveys in 2016 about past 6-month travel and lifetime migration histories, including reasons and locations, and engagement in HIV care defined as (1) discontinuation of care, and (2) history of a treatment interruption among those who remained in care. We estimated associations between mobility and these care engagement outcomes via logistic regression, adjusted for sex, prior mobility, age, region, marital status, household wealth, and education. Results: Among 1081 participants, 56 (5%) reported having discontinued care; among those in care, 104 (10%) reported treatment interruption. Past-year migration was associated with a higher risk of discontinuation of care (adjusted odds ratio [aOR] 1.98, 95% CI 1.08–3.63). In sex-stratified models, the association was somewhat attenuated in women, but remained robust among men. Past-year migration was associated with reduced odds of having a treatment interruption among men (aOR 0.51, 95% CI 0.34–0.77) but not among women (aOR 2.67, 95% CI 0.78, 9.16). Travel in the past 6 months was not associated with discontinuation of care or treatment interruptions. Conclusions: We observed both negative and protective effects of recent migration on care engagement and ART use that were most pronounced among men in this cohort. Migration can break ties to ongoing care, but for men, who have more agency in the decision to migrate, may foster new care and treatment strategies. Strategies that enable health facilities to support individuals throughout the process of transferring care could alleviate the risk of care disengagement.

## 1. Introduction

Human mobility is highly prevalent in sub-Saharan Africa and is a critical aspect of existence and survival. Mobility is important for maintaining social ties and interaction, finding employment and income opportunities, escaping disasters, and avoiding conflicts [1,2,3]. Human mobility, however, may threaten optimal treatment outcomes among persons living with HIV (PLHIV) by compromising retention in care and adherence to antiretroviral treatment (ART) [4], creating vulnerability in this group. This is especially so in sub-Saharan Africa, where mobile individuals are less likely to remain on antiretroviral treatment and achieve viral suppression compared to non-mobile populations [4,5,6].

The definition of mobility remains contentious, with varying measures and definitions having been suggested [7]. In addition, different forms of mobility are yet to be well characterized, such as the drivers of mobility (e.g., work-related and non-work-related mobility), mobility by distance travelled, and differences by sex. Notwithstanding this ongoing debate, research continues to show that different forms of mobility present a wide variety of barriers to care engagement that care recipients must navigate to optimize treatment outcomes. These are partly driven by the stigma around HIV and a fear of disclosure of one’s status [8]. Other barriers to engagement include difficulty finding new/alternative treatment locations, forgetting to bring along ART pills during travel, as well as inflexibility in health systems, which may not accommodate mobile individuals in need of ART refills [9]. On the other hand, mobility could lead to more financial stability or better socio-economic circumstances, and thus improve care engagement via enhancing the ability of mobile individuals to prioritize their health. However, data describing the subtleties of types of mobility and care outcomes, other than qualitative data, are sparse, and therefore this area requires further exploration.

In a population-based cohort of adults living with HIV in Kenya and Uganda, we sought to examine the association between various forms of human mobility with retention in care and antiretroviral treatment (ART) interruptions, and assessed whether these associations differed by sex. We hypothesized that by accounting for gender and different forms of mobility, we would identify both positive and negative associations with engagement in care, which could—in turn—suggest interventions to improve treatment outcomes.

## 2. Methods

### 2.1. Study Design and Sample

We conducted a cross-sectional study among persons living with HIV to examine associations between forms of recent mobility and HIV care engagement. Data are from the baseline visit in a cohort [10,11] of adults sampled from the Sustainable East Africa Research in Community Health (SEARCH) trial (NCT01864603) [12]. SEARCH was a cluster-randomized trial conducted between 2013 and 2017 in Kenya and Uganda designed to test the effect of universal ART (“test and treat”) on HIV incidence and other health outcomes [12]. In 2016, the “Mobility in SEARCH” study enrolled a cohort from 6 control and 6 intervention SEARCH communities via stratified random sampling (defined by gender, HIV status, and prior mobility) to examine the impact of mobility on sexual risk behavior, HIV and sexually transmitted infection incidence, and HIV care engagement [10,11,13,14,15,16,17]. At enrollment, participants answered detailed questionnaires regarding demographics, lifetime migration histories, and overnight travel in the past 6 months. Those who confirmed their HIV status at enrollment completed HIV care engagement questionnaires. This analysis is limited to participants living with HIV who completed the HIV questionnaires.

### 2.2. Study Measures

**Exposures**. Participants who changed their residence in the prior year were classified as having migrated. For overnight travel in the prior 6 months, participants reported the location and reasons for travel to each destination. From these data, we defined three exposures of interest as categorical variables: (1) any overnight travel; (2) travel by distance, categorized as all inter-district (outside their home district (Uganda) or county (Kenya)) or all intra-district (within their home district/county), both, or none; (3) travel reasons, classified as work-related, non-work related, or none. Those who reported both work and non-work travel were classified in the work-related travel group.

**Outcomes**. We defined disengaged from care as individuals who answered “no” to “*Are you currently receiving regular care and treatment for HIV?*” (all had been previously linked to care via SEARCH), or “no” to currently taking ART among those who reported having previously initiated. Those who had not disengaged from care were asked, “*Have you ever missed HIV care appointments, or dropped out of care for a time?*” and those who answered “yes” were classified as having a treatment interruption.

**Covariates**. Because sampling strata for the cohort included sex and prior mobility (spent ≥ 1 month away from the community in the past year at SEARCH baseline), we adjusted all analyses for these indicators. Additionally, we decided a priori to adjust for the following potential confounders: age, region (eastern Uganda, southwestern Uganda, western Kenya), marital status, household wealth (based on a principal components analysis), and education.

### 2.3. Statistical Analysis

We estimated associations between mobility measures and study outcomes via logistic regression, with standard errors accounting for intragroup correlation within SEARCH communities using STATA’s (version 18) clustered sandwich estimator. We built separate models for each mobility metric in association with each study outcome, and report both unadjusted and adjusted analyses. We estimated associations overall and stratified by sex. Because we observed a change in the direction of the odds ratio for treatment interruptions (compared to care engagement) among men after covariate adjustment, we closely examined this model with each covariate separately and described these findings in the results.

### 2.4. Ethical Approvals

This study was approved by the Institutional Review Board at the University of California San Francisco, the Scientific and Ethics Review Unit of the Kenya Medical Research Institute, and the Makerere University School of Medicine Research and Ethics Committee in Uganda. All participants provided written informed consent.

## 3. Results

Of 1119 participants identified as living with HIV at the SEARCH baseline, 1081 (97%) confirmed their HIV status at enrollment into the cohort study and are included in this analysis. Excluded participants (n = 38, i.e., those who did not confirm their HIV status at enrollment into the cohort) were more likely to be male, single, and to report no mobility in the prior 6 months (data not shown). Among included participants, the median age among males was 43 years (interquartile range (IQR): 36–51) and among females was 40 (IQR: 32–48) (Table 1). Most men were married (92%), while just over half of women were married (58%). More women reported overnight travel in the prior 6 months (58%) compared to men (48%), while more men (9%) than women (6%) migrated in the prior year. The vast majority of women’s mobility was non-work-related, with only 4% reporting travel for work, while men reported travel for both reasons with similar frequency (24%). Intra-district travel (28%) was more common than inter-district (15%), particularly among women, with 33% reporting solely intra-district travel.

Overall, 56 (5%) reported having discontinued care, with no difference between men and women. Lower household wealth, a marital status of being single, and past-year migration were associated with a higher risk of discontinuation of care, while older participants and those from Uganda had a lower risk of discontinuation. Among those in care, 104 (10%) reported a treatment interruption, including 15% of men and 6% of women. Those with more than a primary education, those living in southwestern Uganda communities, and those with past-year migration had a higher risk of a treatment interruption, while widowed or separated participants and those from east Uganda had a lower risk.

### 3.1. Association between Mobility and Discontinuation of Care

Past-year migration was associated with three times the odds of discontinuation of care in an unadjusted analysis (OR 3.01, 95% CI 1.80–5.05), which was modestly attenuated after adjusting for demographic characteristics (OR 1.98, 95% CI 1.08–3.63) (Table 2). This association was similar in men and women, though only remained statistically significant among men in adjusted analysis (OR 2.05, 95% CI 1.15–3.67). Past 6-month travel, regardless of distance or reason, was not associated with discontinuation of care in adjusted analyses.

### 3.2. Association between Mobility and Treatment Interruptions

Past-year migration was associated with twice the odds of having a treatment interruption in unadjusted analysis (OR 1.98, 95% CI 1.01–3.87), but this association was attenuated to the null in the adjusted model. Notably, in adjusted models, past-year migration was predictive of having a treatment interruption among men (OR 0.51, 95% CI 0.34–0.77) but not among women (OR 2.67, 95% CI 0.78, 9.16). Although the crude association among men was null (OR 1.20, 95% CI 0.70, 2.06), this finding was in the opposite direction of the association with care engagement, so we carefully investigated which covariate drove this change in the adjusted model. We found that region was the primary driver. Specifically, in Kenya, among men who reported past-year migration, none reported a treatment interruption. In east Uganda, there were no past year migrations among men. In Southwestern Uganda, 39% of men who recently migrated and 40% of men who did not migrate reported a treatment interruption. We could not run regression models stratified by each region given the zero cells in Kenya and east Uganda, but in an adjusted model within southwestern Uganda alone, the protective association with recent migration held, though was somewhat attenuated (OR 0.68, 95% CI 0.48, 0.98). We found that age was the primary driver of this difference between crude and adjusted models within the region of southwestern Uganda, as younger age is associated with both treatment interruptions and recent migration in this cohort. Travel in the past 6 months, regardless of distance or reason, was not associated with having a treatment interruption overall or stratified by sex.

### 3.3. Reasons for Poor Care Engagement

Among participants who had dropped out of care, the most common barriers reported included fear of disclosure when attending clinic (36%), feeling well and not perceiving a need for treatment (29%), and not being able to afford transit to the clinic (13%) (Table 3). Among those who reported having a treatment interruption, the most common barrier was long wait times interfering with work (42%) closely followed by not being able to afford transit to the clinic (38%).

## 4. Discussion

In a large population-based cohort of adults living with HIV from Kenya and Uganda, we observed strong associations between recent migration and HIV care utilization, with important differences in terms of sex. For men, recent migration was associated with a significantly higher risk of discontinuation of care, yet, among those who remained in care, those who recently migrated were less likely to report a treatment interruption. For women who recently migrated, the risk of both discontinuation and temporary interruptions was elevated, but neither association was significant after adjustment for relevant covariates. We did not observe associations between recent overnight travel and care engagement, regardless of the reason for or distance of travel.

Our analysis incorporated more nuanced measures of mobility than most prior work in this area. We hypothesized that with these nuanced measures, we would find varied associations between different forms of mobility and care utilization, both higher and lower risk, as we recently observed when examining mobility and ART treatment adherence [17]. However, the majority of our estimates were either close to the null or had wide confidence intervals. We have considered three possible explanations for these null findings. First, both the discontinuation of care and treatment interruptions were rare in this population, which may have impacted our ability to detect significant associations. Second, the median age in our cohort was 43 years, most participants were over 30, and 92% of men in the sample were married. While mobility may influence access to ongoing care for all populations, our sample may be more resilient to the disruptions that mobility presents than among young, unmarried adults [18]. Third, the questions regarding care discontinuation and treatment interruptions were not time-bound, and those who reported either outcome may have been referencing events that were long past and thus less likely to be impacted by past 6-month mobility.

Still, consistent with prior studies [5,19,20,21], we did observe a strong association between recent migration and care discontinuation, particularly for men. Other studies have included different populations—such as postpartum women in South Africa; [20,22] and only men in Malawi [21]—but the finding that mobility is associated with care discontinuation tends to be consistent across populations and settings. Relocating may require transferring care facilities or making special arrangements to travel back to one’s prior home to maintain care. Even in the optimal scenario with a formal transfer letter, the initiative to engage with a new provider and to navigate transit to a new clinic requires persistence and a greater commitment than following old routines. On the other hand, we were surprised that recent migration was associated with a lower risk of treatment interruptions for men who were in care at the time of this study. With a detailed assessment of our adjusted model, we found this protective association was robust. We previously found that short-duration work-related travel among men was associated with improved ART adherence in this cohort [17]. In this current analysis, the numbers were too small to categorize recent migration by reason; however, men’s mobility is more often linked to income and privilege, which could facilitate continuity of care and pill-taking [23]. Alternatively, given that HIV stigma was the most commonly reported barrier to staying in care, men who migrate and manage to successfully transfer care may experience some freedom from stigma with the anonymity that comes with a new setting.

In our assessment of reasons for discontinuation or treatment interruptions, HIV stigma, long travel or wait times that interfere with work, and the expense of transit were the major drivers of these poor outcomes. These data reinforce the importance of differentiated care options to alleviate the burden and costs of travel, time spent attending clinic visits and obtaining refills, and concerns about stigma when attending the clinic. Moreover, given the association between migration and care discontinuation, coupled with these concerns about transit expenses, novel supportive interventions that minimize the burden of care navigation are needed for this population.

Our study has some limitations. First, as mentioned above, our questions about care engagement were not time-bound. This was informed by evidence from previous studies showing that prior HIV treatment interruption was a predictor of future treatment interruption [24,25], an outcome of interest in our analysis. For treatment interruptions, participants were expected to interpret this question to include long-past interruptions. However, we did observe a high frequency of reported interruptions in Uganda, despite having a low frequency of discontinuation of care, which suggests the question may have been interpreted differently than in Kenya. However, for discontinuation of care, even if care had been dropped for a long duration, by definition, those who dropped care had not re-engaged and thus had a current challenge accessing care at the time of the interview. Our findings differ slightly from another study in East Africa, which found that cross-border migrants were as likely to be in care as non-migrants, but they were less likely to be virally suppressed [26]; this may suggest that we captured longer histories of treatment interruption as opposed to more recent gaps. Second, viral loads were not collected within the mobility cohort study; thus, we did not include this important treatment outcome. We considered examining viral loads collected within SEARCH, but because study visits were not aligned between the two studies, we determined the measures to be too far apart in time to perform a meaningful assessment. Third, 38 individuals (3% of the sample) could not be included in the analysis due to not confirming their status and these individuals differed by sex, marital status, and prior mobility from the larger sample. However, we think selection bias is unlikely both because we adjusted for each of these factors in multivariable models and because the number excluded is likely not large enough to meaningfully influence the results. Finally, sparse data in our outcomes may have limited our ability to detect significant associations between mobility and outcomes, particularly among women alone.

In conclusion, recent migration was strongly associated with the discontinuation of care in this cohort—more so for men than for women. Strategies that enable facilities to anticipate changes of residence, particularly for men, could ensure ongoing support throughout the transfer of care, from preparation through an established link with a new facility, which could alleviate the risk of disengagement. Our study thereby suggests interventions for helping re-engage mobile populations living with HIV into care.

## Figures and Tables

**Table 1:** Participant characteristics.

	Men	Women	Total
	n = 499	n = 582	n = 1081
Region			
Kenya	272 (55%)	308 (53%)	580 (54%)
Uganda east	93 (19%)	132 (23%)	225 (21%)
Uganda southwest	134 (27%)	142 (24%)	276 (26%)
Current age	43 (36–51)	40 (32–48)	41 (34–50)
Marital status			
Married	457 (92%)	336 (58%)	793 (73%)
Single	19 (4%)	26 (4%)	45 (4%)
Widowed/divorced/separated	23 (5%)	220 (38%)	243 (22%)
Education			
None	43 (9%)	103 (18%)	146 (14%)
Primary	299 (60%)	362 (62%)	661 (61%)
More than primary	155 (31%)	115 (20%)	270 (25%)
Missing	2 (0%)	2 (0%)	4 (0%)
Household wealth, lowest quintile	97 (19%)	105 (18%)	202 (19%)
Occupation categories			
Farming	270 (54%)	352 (60%)	622 (58%)
Fishing/fish market	84 (17%)	56 (10%)	140 (13%)
Other	129 (26%)	148 (25%)	277 (26%)
Missing	16 (3%)	26 (4%)	42 (4%)
Travel work or other reasons, past 6 months			
None	259 (52%)	243 (42%)	502 (46%)
Work	121 (24%)	23 (4%)	144 (13%)
Non-work only	119 (24%)	316 (54%)	435 (40%)
Main travel distance, past 6 months			
None	259 (52%)	243 (42%)	502 (46%)
All inter-district	84 (17%)	79 (14%)	163 (15%)
All intra-district	108 (22%)	194 (33%)	302 (28%)
Both inter- and intra-district	48 (10%)	66 (11%)	114 (11%)
Any migration, past year			
No	454 (91%)	548 (94%)	1002 (93%)
Yes	45 (9%)	34 (6%)	79 (7%)
Prior mobility at SEARCH baseline			
No	301 (60%)	362 (62%)	663 (61%)
Yes	198 (40%)	220 (38%)	418 (39%)

**Table 2:** Associations between recent mobility and HIV care engagement (discontinuation of care and treatment interruptions).

	Total n = 1081				Men n = 499				Women n = 582			
	Unadjusted		Adjusted		Unadjusted		Adjusted		Unadjusted		Adjusted	
	**Discontinued care**											
	OR (95% CI)	*p*-value	OR (95% CI)	*p*-value	OR (95% CI)	*p*-value	OR (95% CI)	*p*-value	OR (95% CI)	*p*-value	OR (95% CI)	*p*-value
Any past-year migration	3.01 (1.80, 5.05)	0.000	1.98 (1.08, 3.63)	0.03	2.71 (1.35, 5.45)	0.005	2.05 (1.15, 3.67)	0.02	3.46 (1.26, 9.48)	0.02	1.64 (0.61, 4.42)	0.33
Any past 6-month travel	1.60 (0.68, 3.74)	0.28	0.96 (0.45, 2.03)	0.91	1.18 (0.45, 3.12)	0.74	0.88 (0.28, 2.73)	0.82	2.14 (0.83, 5.53)	0.12	1.01 (0.46, 2.20)	0.98
Past 6 m travel distance												
None	(ref *)		(ref)		(ref)		(ref)		(ref)		(ref)	
All inter-district	0.92 (0.32, 2.65)	0.88	0.64 (0.22, 1.84)	0.41	1.30 (0.35, 4.88)	0.70	0.98 (0.22, 4.48)	0.98	0.38 (0.04, 3.81)	0.41	0.22 (0.02, 2.10)	0.19
All intra-district	2.08 (0.83, 5.24)	0.12	1.16 (0.53, 2.54)	0.71	1.21 (0.36, 4.11)	0.76	0.90 (0.23, 3.59)	0.89	3.00 (1.19, 7.55)	0.02	1.30 (0.63, 2.66)	0.47
Both	1.34 (0.62, 2.87)	0.45	0.86 (0.46, 1.63)	0.65	0.89 (0.38, 2.11)	0.80	0.62 (0.32, 1.20)	0.15	1.90 (0.57, 6.26)	0.29	1.09 (0.35, 3.35)	0.89
Past 6 m travel reasons												
None	(ref)		(ref)		(ref)		(ref)		(ref)		(ref)	
Work	1.80 (0.64, 5.07)	0.27	1.20 (0.43, 3.40)	0.73	1.46 (0.48, 4.47)	0.51	1.32 (0.34, 5.11)	0.69	2.80 (0.66, 11.82)	0.16	1.10 (0.21, 5.76)	0.91
Non-work only	1.53 (0.64, 3.65)	0.34	0.87 (0.41, 1.85)	0.72	0.90 (0.37, 2.18)	0.82	0.57 (0.20, 1.63)	0.30	2.09 (0.81, 5.41)	0.13	1.00 (0.45, 2.23)	0.99
	**Treatment interruption**											
	OR (95% CI)	*p*-value	OR (95% CI)	*p*-value	OR (95% CI)	*p*-value	OR (95% CI)	*p*-value	OR (95% CI)	*p*-value	OR (95% CI)	*p*-value
Any past-year migration	1.98 (1.01, 3.87)	0.047	1.15 (0.59, 2.23)	0.68	1.20 (0.70, 2.06)	0.50	0.51 (0.34, 0.77)	0.001	3.82 (1.16, 12.55)	0.03	2.67 (0.78, 9.16)	0.12
Any past 6-month travel	0.95 (0.50, 1.83)	0.89	0.86 (0.63, 1.17)	0.34	0.91 (0.49, 1.67)	0.76	0.91 (0.52, 1.56)	0.72	1.45 (0.72, 2.94)	0.30	0.86 (0.53, 1.39)	0.53
Past 6 m ravel distance												
None	(ref)		(ref)		(ref)		(ref)		(ref)		(ref)	
All inter-district	1.71 (0.88, 3.34)	0.11	0.95 (0.70, 1.31)	0.78	1.57 (0.85, 2.91)	0.15	0.77 (0.43, 1.39)	0.39	2.33 (0.88, 6.13)	0.09	1.46 (0.78, 2.72)	0.23
All intra-district	0.67 (0.34, 1.32)	0.25	0.82 (0.50, 1.34)	0.42	0.52 (0.23, 1.17)	0.11	1.05 (0.46, 2.37)	0.91	1.36 (0.62, 2.98)	0.45	0.77 (0.36, 1.67)	0.51
Both	0.69 (0.24, 1.99)	0.49	0.70 (0.31, 1.58)	0.39	0.80 (0.30, 2.14)	0.66	1.25 (0.51, 3.08)	0.62	0.68 (0.14, 3.24)	0.63	0.37 (0.08, 1.79)	0.22
Past 6 m travel reasons												
None	(ref)		(ref)		(ref)		(ref)		(ref)		(ref)	
Work	1.98 (0.98, 4.02)	0.06	1.19 (0.59, 2.39)	0.63	1.29 (0.65, 2.55)	0.47	1.17 (0.43, 3.22)	0.76	3.39 (0.91, 12.69)	0.07	2.48 (0.61, 10.05)	0.20
Non-work only	0.66 (0.31, 1.42)	0.29	0.68 (0.46, 1.02)	0.06	0.57 (0.22, 1.45)	0.24	0.61 (0.26, 1.44)	0.26	1.32 (0.62, 2.84)	0.47	0.77 (0.47, 1.28)	0.31

* Ref stands for reference or reference group.

**Table 3:** Barriers to care among those reporting discontinuation of care or a treatment interruption.

IF DROPPED CARE: Can You Tell Me the Main Reasons You’re Not Receiving care Currently? (n = 56)		
	n	% reporting yes
HIV/AIDS stigma: fear of disclosure at clinic/at home	20	36%
Felt well and did not think s/he needs ARVs	16	29%
Can’t afford transport to clinic	7	13%
Visits to clinic interfere with work/wait times are too long	4	7%
Afraid of side effects	3	5%
Treated poorly by clinic staff in the past	2	4%
Tired of taking ARVs/Don’t want to have to take pills for the rest of his/her life	2	4%
Seeking a spiritual alternative/traditional medicine/prayer	2	4%
Not enough food to continue ARVs/afraid to start because food insecure	1	2%
Other	4	7%
IF TREATMENT INTERRUPTION—What are the main barriers you face, to being able to make appointments? (n = 104)		
	n	%
Visits to clinic interfere with work/wait times are too long	44	42%
Can’t afford transport to clinic	39	38%
Treated poorly by clinic staff in the past	11	11%
HIV/AIDS stigma: fear of disclosure at clinic/at home	9	9%
Feels well and does not think s/he needs HIV care	9	9%
Tired of taking ARVs/Don’t want to have to take pills for the rest of his/her life	3	3%
Not enough food to continue ARVs/afraid to start because food insecure	3	3%
Seeking a spiritual alternative/traditional medicine/prayer	2	2%
Other	37	36%

## Data Availability

Data is available upon reasonable request from the study lead C.S.C.
